# A rare case of *Pantoea* bacterial species infection: A new mimicker in the dermatology office

**DOI:** 10.1016/j.jdcr.2024.01.019

**Published:** 2024-02-23

**Authors:** Benjamin Kahn, Shannon Hart, Heather Ivy Hensley, Marcus Goodman, Kristopher McKay

**Affiliations:** a Goodman Dermatology, Roswell, Georgia; b Edward Via College of Osteopathic Medicine, Auburn, Alabama; c SkinPath Solutions, Smyrna, Georgia

**Keywords:** bacterial infection, Bowen disease, indole-3-acetic acid, Pantoea agglomerans, squamous cell carcinoma

## Introduction

*Pantoea agglomerans*, previously *Enterobacter agglomerans*, is a gram-negative aerobic bacillus frequently isolated from plants, soil, food, and feces of humans and animals.^1^ This yellow-pigmented organism belongs to the Enterobacteriaceae family and typically affects immunocompromised individuals in the setting of a wound infection or hospital-acquired contamination.^2^

*P agglomerans* typically results in soft tissue or bone and joint infections secondary to penetrating trauma by wooden material such as trees; however, there are only a few cutaneous cases reported in literature.^3^^,^^4^ Several authors have postulated that the secretion of auxins, such as indole-3-acetic acid, by *P agglomerans*, has allowed this gram-negative bacterium to modulate host cell defenses by invading plant cytosols, facilitating successful colonization and growth.^5^ Several reports have noted the occurrence of this species on several plants, such as apple and pear trees, secondary to blossom-to-blossom spread, which is favored in warm, dry environments.^6^

## Case report

**Fig 1:**
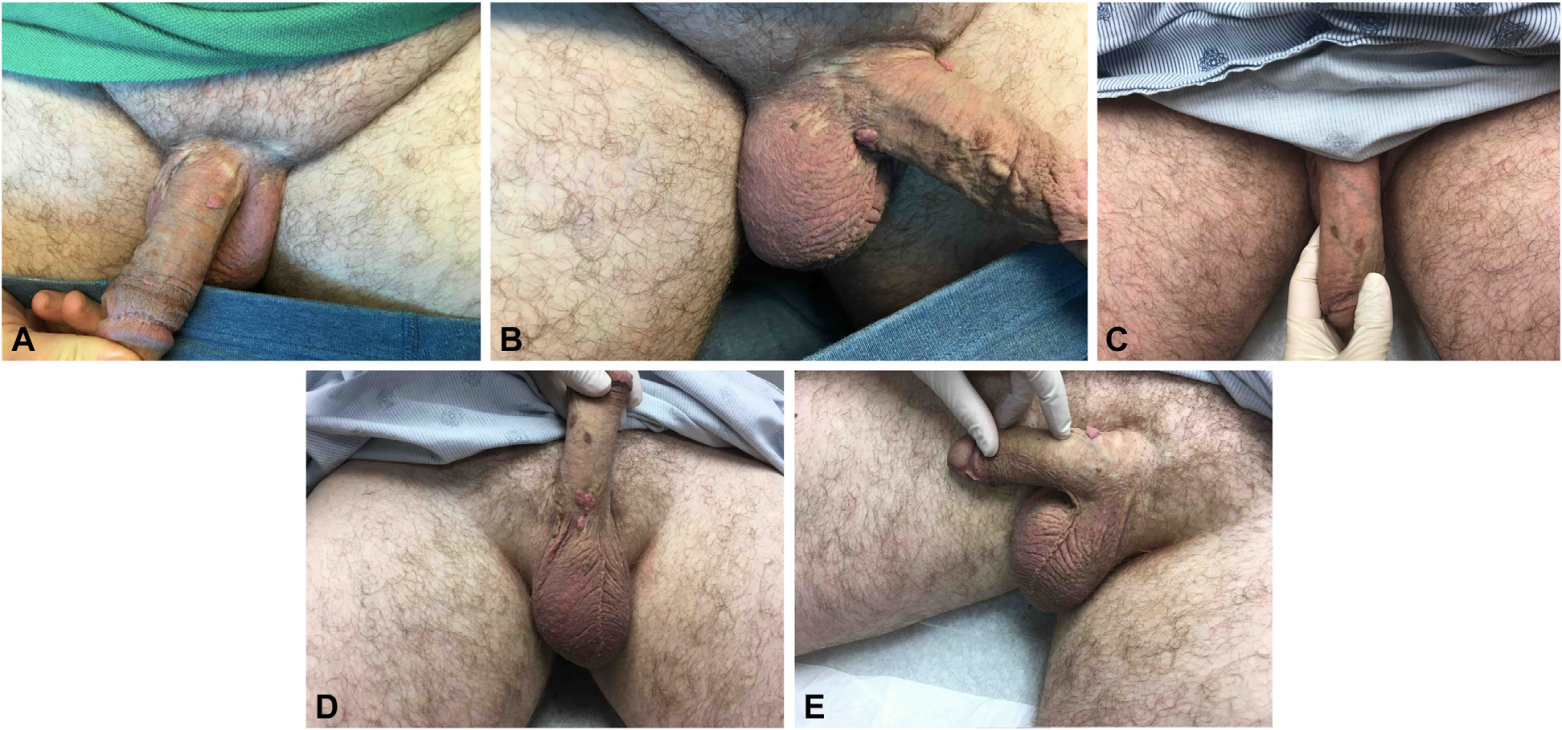
**A, B,** Patient of interest presented in 2018 with raised, flesh-colored papules on the ventral surface of the penile shaft demonstrating high-risk human papillomavirus 16 DNA strain. **C,** In 2019, patient developed more flesh-colored lesions, highlighting the reoccurrence of genital warts on the shaft of the penis. **D, E** In 2022, the patient continued to develop genital warts on the ventral and dorsal shaft of the penis.

A 54-year-old man, with a history of genital warts, presented to the clinic with a mildly painful and pruritic grayish-red macerated plaque with ulceration and erythematous borders localized on the penis for approximately 3 weeks. Before presentation, the patient had been experiencing genital warts on the ventral and dorsal shaft of the penis and right scrotum from 2017 to 2021 (Fig 1, *A-E*). Biopsies of the genital warts were obtained after failed treatment with topical imiquimod and liquid nitrogen cryotherapy, which demonstrated high-risk human papillomavirus (HPV) 16 DNA strain. Treatment was then modified to multiple rounds of liquid nitrogen cryotherapy and ozenoxacin cream 1% with improvement.

**Fig 2:**
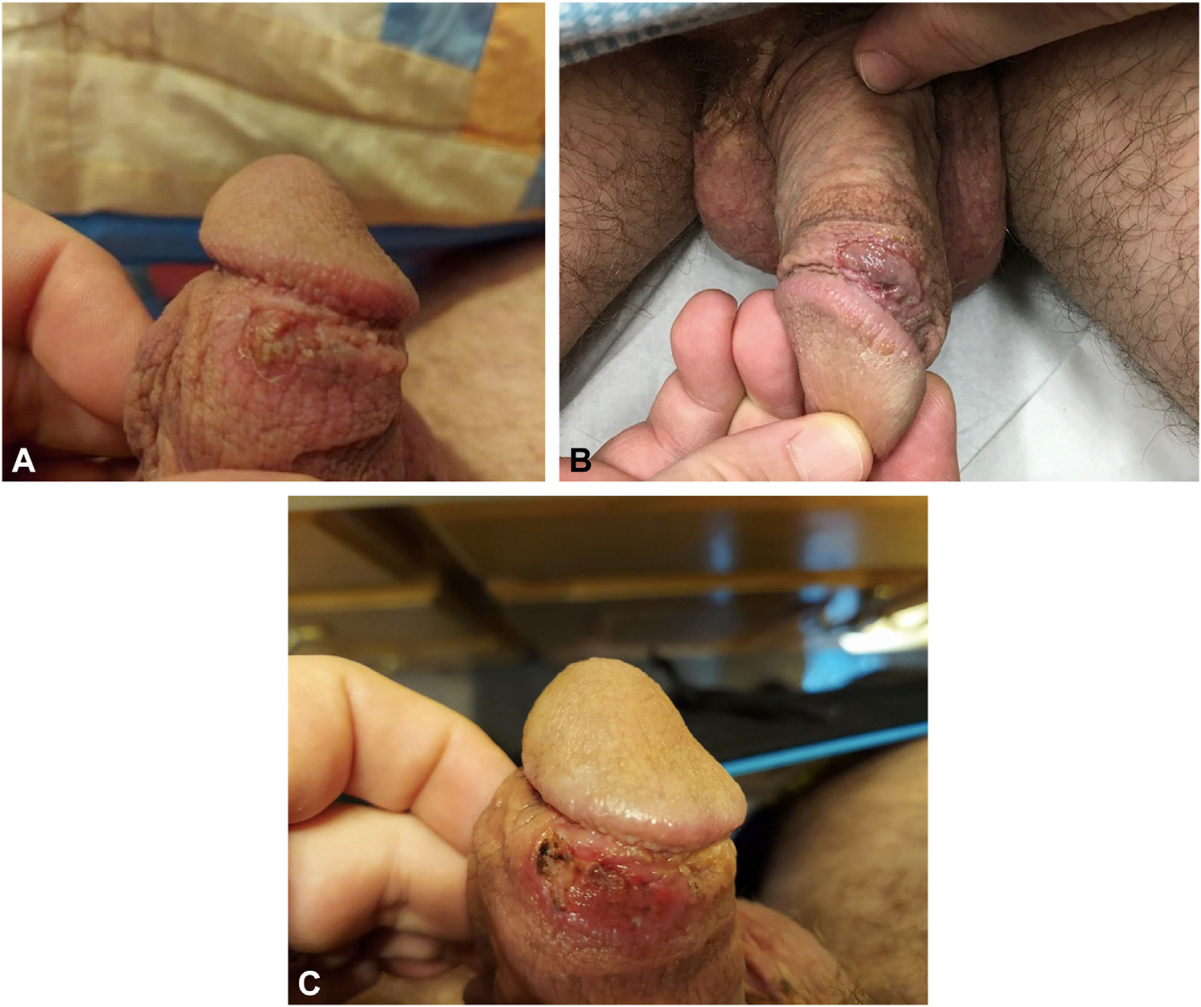
**A,** In September 2022, picture taken at home by patient demonstrating a mildly painful and pruritic grayish-red macerated plaque with ulceration and erythematous borders on the penis. **B,** Lesion increased in sized over a few weeks and presented to the dermatology clinic. **C,** Lesion postbiopsy.

In early September 2022, the patient visited an urgent care facility complaining of a new and unusual, painful lesion at the right distal dorsal penile shaft, which was treated as a potential herpes outbreak (Fig 2, *A*). A culture of the lesion was obtained, and the patient was then started on valacyclovir 1 g twice a day for 7 days and mupirocin ointment. The culture resulted negative for viruses including herpes and monkeypox, and the lesion continued to increase in size.

**Fig 3:**
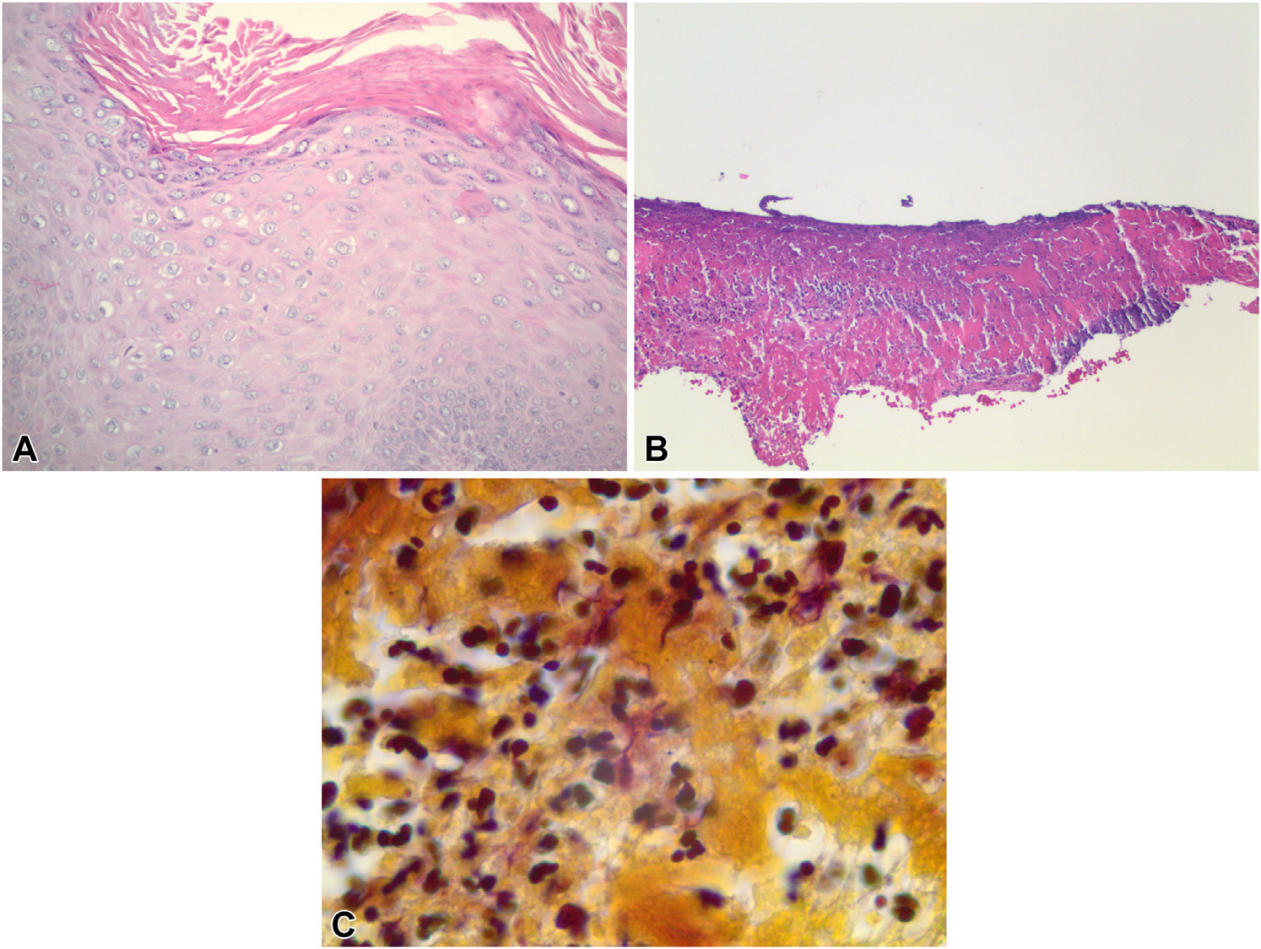
**A,** Routine stain. Biopsy obtained from lesion localized on the penis of the patient. Section revealed hyperkeratosis, acanthosis, and koilocytic change per pathology report. **B,** Hematoxylin-eosin stain (original magnification: ×10). Conventional sections show an ulcer with associated necroinflammatory changes and neutrophil collections. **C,** Gram stain (original magnification: ×100). Gram stain used was Brown and Brenn method. Demonstrated occasional gram-negative rods amid neutrophil collections, just deep to the ulcer base.

**Fig 4:**
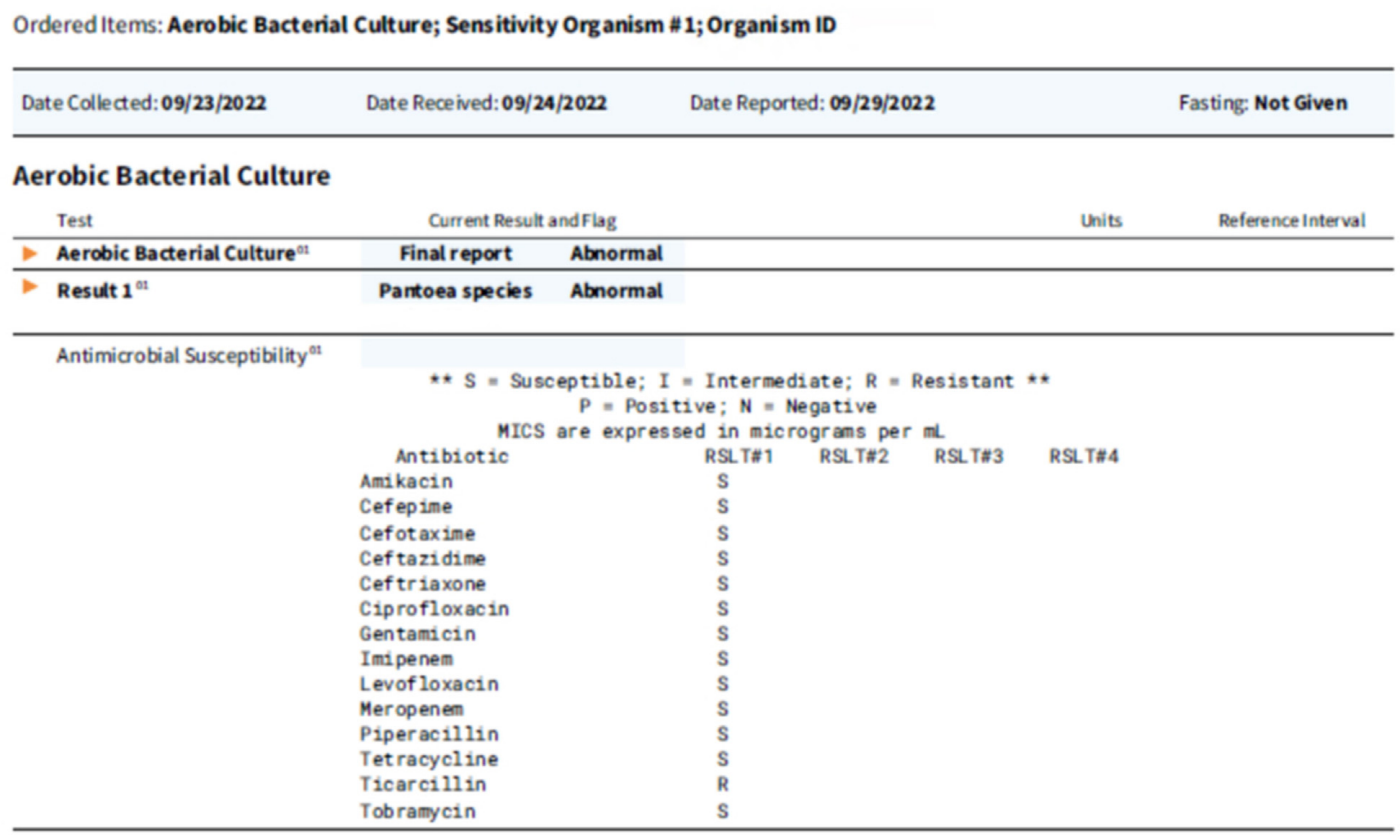
Aerobic bacteria culture report demonstrating growth of *Pantoea* species.

Secondary to unresolving symptoms, the patient visited a dermatology office in late September 2022, and a shave biopsy of the lesion was performed (Fig 2, *B*). Considered in the differential diagnosis were condyloma acuminatum with underlying ulceration, herpes simplex virus type 1 or 2 ulcerated lesion, bowenoid papulosis, chancroid, syphilis, and lymphogranuloma venereum, malignant transformation to squamous cell carcinoma of the penis, Erythroplasia of Queyrat, or Bowen disease (Fig 2, *C*) secondary to the patient’s long-standing history of HPV 16 genital warts. While completing an extensive history and physical examination, the patient admitted to frequent gardening outside with apple, pear, and oak trees at his home. Results of the biopsy (Fig 3, *A*), hematoxylin-eosin stain (Fig 3, *B*), gram stain (Brown and Brenn method) (Fig 3, *C*) and tissue culture (Fig 4) were negative for fungus, viruses, sexually transmitted infections, and malignancy, but positive for gram-negative rods on tissue gram stain. This unexpected finding correlated well with the patient’s positive tissue culture of the biopsied lesion (Fig 4) which supported the diagnosis of *Pantoea* species bacterial infection with ulceration and necrosis of the penis.

The patient was prescribed triamcinolone 0.025% cream twice a day (2 weeks on, 1 week off, repeat as needed), topical mupirocin 2% ointment twice a day, and doxycycline 100 mg taken by mouth twice a day for 7 days. Approximately 2 weeks later, the patient contacted the office stating the lesion had been improving on current treatment and was advised to continue the use of prescribed topicals until the lesion was completely healed. Three months later, the patient returned to the clinic for a full body skin examination and the site of the lesion was examined, which demonstrated complete resolution.

## Discussion

Infections caused by *P agglomerans* are rare and primarily occur in humans secondary to penetrating wound or in a hospital-acquired setting, and is the most common isolated species of this genus in humans.^7^ This gram-negative bacterium, primarily found in agriculture and the environment, usually presents as joint or bone infections except in this unusual case where *P agglomerans* manifested cutaneously.

Plant pathogens are able to manipulate their hosts to facilitate the growth and production of disease. Several plant pathogens, including *P agglomerans*, secrete auxins, which inhibit host defenses and promote pathogen development.^3^ The *Pantoea* species has demonstrated the production of an auxin named indole-3-acetic acid, known to aid in priming and establishing a bacteria-plant relationship within the root system architecture.^8^ The exact mode of transmission is unknown for our patient; however, he likely was inoculated by this bacterium in his backyard during frequent exposure while gardening and handling apple and pear trees associated with *P agglomerans* then going to the restroom.

Indole-3-acetic acid is a hazardous substance, according to the Occupational Safety and Health Administration. Individuals should avoid skin contact secondary to inflammation and the accentuation of preexisting dermatitis. Globally Harmonized System for Classification and Labeling of Chemicals categorizes indole-3-acetic acid as a category 2 compound causing skin irritation and corrosion, which supports the ulceration and necrosis on the biopsy report from this patient. Treatment has been successful with tetracyclines in previously reported cases.^8^ Our patient was treated with a 7-day course of doxycycline 100 mg twice a day, triamcinolone 0.025% cream, and topical mupirocin ointment daily.

As authors, we believe this case is significant due to *P agglomerans* ability to clinically mimic several diseases such as HPV, herpes simplex virus, chancroid, lymphogranuloma venereum, bowenoid papulosis, syphilis as well as squamous cell carcinoma of the penis. squamous cell carcinoma of the penis typically involves the glans penis, prepuce, or the urethral meatus and presents itself as a red, well-defined, slightly raised smooth plaque, often with HPV 16 and 18 coinfections.^9^ The biopsy and hematoxylin-eosin stain demonstrated unremarkable findings of ulceration with necrosis (Fig 3, *A, B*) and the gram stain displayed gram-negative rods amid neutrophil collections (Fig 3, *C*). These findings became a pivotal point in our case—subsequently eliminating several differentials. The clinical history and all laboratory findings support our diagnosis of genital inoculation with *P agglomerans*. More research for literature is needed to fully elucidate this rare entity and its impact on humans and infectious diseases. Similar to the “great mimickers” of the past, move over syphilis, *P agglomerans* fits the new bill and should be considered by farmers, gardeners, botanists, plant handlers, loggers, and florists for unusual lesions.

## Conflicts of interest

None disclosed.
